# Social Determinants of Health Screening by Preclinical Medical Students During the COVID-19 Pandemic: Service-Based Learning Case Study

**DOI:** 10.2196/32818

**Published:** 2022-01-17

**Authors:** Tara Herrera, Kevin P Fiori, Heather Archer-Dyer, David W Lounsbury, Judith Wylie-Rosett

**Affiliations:** 1 Office of Student Research Albert Einstein College of Medicine Bronx, NY United States; 2 Department of Family and Social Medicine Albert Einstein College of Medicine Bronx, NY United States; 3 Department of Epidemiology and Population Health Albert Einstein College of Medicine Bronx, NY United States

**Keywords:** social determinants of health, service-based learning, telehealth, preclinical education, screening, referral, community health workers, determinant, medical student, case study, service, preparation, pilot, feasibility, training, assessment, needs, electronic health record, questionnaire

## Abstract

**Background:**

The inclusion of social determinants of health is mandated for undergraduate medical education. However, little is known about how to prepare preclinical students for real-world screening and referrals for addressing social determinants of health.

**Objective:**

This pilot project’s objective was to evaluate the feasibility of using a real-world, service-based learning approach for training preclinical students to assess social needs and make relevant referrals via the electronic medical record during the COVID-19 pandemic (May to June 2020).

**Methods:**

This project was designed to address an acute community service need and to teach preclinical, second-year medical student volunteers (n=11) how to assess social needs and make referrals by using the 10-item Social Determinants of Health Screening Questionnaire in the electronic health record (EHR; Epic platform; Epic Systems Corporation). Third-year medical student volunteers (n=3), who had completed 6 clinical rotations, led the 2-hour skills development orientation and were available for ongoing mentoring and peer support. All student-patient communication was conducted by telephone, and bilingual (English and Spanish) students called the patients who preferred to communicate in Spanish. We analyzed EHR data extracted from Epic to evaluate screening and data extracted from REDCap (Research Electronic Data Capture; Vanderbilt University) to evaluate community health workers’ notes. We elicited feedback from the participating preclinical students to evaluate the future use of this community-based service learning approach in our preclinical curriculum.

**Results:**

The preclinical students completed 45 screening interviews. Of the 45 screened patients, 20 (44%) screened positive for at least 1 social need. Almost all of these patients (19/20, 95%) were referred to the community health worker. Half (8/16, 50%) of the patients who had consultations with the community health worker were connected with a relevant social service resource. The preclinical students indicated that project participation increased their ability to assess social needs and make needed EHR referrals. Food insecurity was the most common social need.

**Conclusions:**

Practical exposure to social needs assessment has the potential to help preclinical medical students develop the ability to address social concerns prior to entering clinical clerkships in their third year of medical school. The students can also become familiar with the EHR prior to entering third-year clerkships. Physicians, who are aware of social needs and have the electronic medical record tools and staff resources needed to act, can create workflows to make social needs assessments and services integral components of health care. Research studies and quality improvement initiatives need to investigate how to integrate screening for social needs and connecting patients to the appropriate social services into routine primary care procedures.

## Introduction

The social determinants of health focus on “the conditions in which people are born, grow, live, work and age” and include employment, food security, housing security, access to health care, and transportation as potential contributors to poor health outcomes [1]. Health systems are struggling with how to improve health equity and train care providers to assess social determinants of health and make appropriate referrals in clinical practice [2-10]. The need for social determinants of health screening increased dramatically with the advent of the COVID-19 pandemic in 2020 [11-15]. As stay-at-home orders were instituted, the unemployment rate rose to 20.4% in the New York epicenter by June 2020 [15].

The impact of COVID-19 on health care and medical student training in the United States was dramatic. Health care providers were transferred from outpatient care to inpatient care to manage the influx of gravely ill patients with COVID-19. The Association of American Medical Colleges (AAMC) recommended that medical students be removed from all in-person clinical activities [14]. This left medical students with time for pursuing learning opportunities that do not involve in-person clinical activities.

The AAMC has stressed the importance of service orientation as a core competency that strengthens medical school and residency applications. Community-based service learning activities can facilitate the development of competencies, such as cultural competence and teamwork as well as service orientation, that are needed to improve health equity. The AAMC website suggests that both undergraduate and graduate medical education programs are looking for applicants who “demonstrate a desire to help others and sensitivity to others’ needs and feelings; demonstrate a desire to alleviate others’ distress; [and] recognize and act on [their] responsibilities to society; locally, nationally, and globally” [16]. The motivation to become a physician frequently comes from a desire to help people. Taking a service year prior to medical school is recommended by the AAMC as a way to make a positive impact on people though work. AAMC-suggested activities for a gap service year have included tutoring children, caring for older adults, supporting veterans, aiding people who are homeless, or helping communities recover from natural disasters. These AAMC-suggested community-based service learning activities generally involve in-person contact. The AAMC did not issue guidance for alternative service activities for medical students, who suddenly had gap time after being removed from in-person clinical activities. However, telehealth can provide opportunities for students to participate in community-based service learning that does not involve in-person clinical activities.

This case study describes the telehealth activities of medical student volunteers who used a social needs screening questionnaire and community health worker referrals to provide patient assistance in a primary care practice setting during the height of the COVID-19 pandemic crisis in New York. The objective of our case study was to evaluate the feasibility of using real-world, service-based learning to teach preclinical medical students how to interview patients by telephone, assess social needs, make appropriate referrals, and enter relevant information into patients’ electronic health records (EHRs).

## Methods

### Setting

This case study describes our work with a primary care practice that provides safety net services as a federally qualified health center and serves as a teaching site within a large urban health system. This health center provides clinical services related to family medicine, internal medicine, pediatrics, obstetrics and gynecology, psychiatry, mental health, and social services and has primary care teams that include community health workers [17]. These services are included as part of the clinical rotations for medical students, and this health center is 1 of the 20 primary care practice teaching sites located in the Bronx and Westchester County, New York.

### Patient Population

The medical director identified patients (n=53) for social determinants of health telephone screening based on the following criteria: patients aged ≥50 years, those who were due for colorectal cancer screening (via colonoscopy), and those who did not have insurance. Due to the acuity of the pandemic crisis and the need to screen and intervene on the needs of a large patient population, the contact list provided was based on readily available clinic data (eg, patients due for colonoscopy), and the medical director focused on patients without health insurance for outreach purposes.

### Role of Medical Student Volunteers

Preclinical, second-year medical student volunteers (n=11) were recruited via email. Third-year medical student volunteers (n=3), who had completed 6 clinical rotations, developed and led a 2-hour skills development training workshop for the preclinical second-year students. The workshop PowerPoint slides are in Multimedia Appendix 1. The learning objectives included being able to (1) navigate the Epic platform (Epic Systems Corporation) EHR, (2) screen for social needs, and (3) refer patients to the appropriate care providers. If potential domestic violence was a concern for a patient, they were referred to a social worker; patients with other social needs were referred to the community health worker. The training addressed how to sensitively approach patients about social needs screening. The third-year medical students provided ongoing mentoring, peer support, and guidance (ie, they answered questions) for contacting patients via telephone, conducting patient interviews, and documenting the Social Determinants of Health Screening Questionnaire assessments and related referrals via the Epic portal.

### Preclinical Training: Social Determinants of Health and Community-Based Service Learning

Preclinical medical students have varying degrees of exposure to opportunities for learning about social determinants of health–related topics and developing skills for addressing socially determined health disparities. Exposure is provided via the medical school curriculum, and skills can be acquired by volunteering in community-based service learning projects. Embedded in the first 2 years of the medical school curriculum is the *Introduction to Clinical Medicine* longitudinal course*.* The topics addressed in this course include patient health literacy, HIV, social determinants of health, and substance abuse. Interviewing skills for obtaining a sexual history are taught via practice mock interviews with standardized patients. The medical school volunteer opportunities are available through the *Community-Based Service Learning* program, which is comprised of a network of student-initiated projects, including the sponsoring of a community garden and a wide variety of community education projects. Students also have the option of volunteering at the Einstein Community Health Outreach clinic, which is a largely medical student–run free clinic that aims to provide quality care to uninsured patients. Preclinical medical student volunteers at the Einstein Community Health Outreach clinic serve a variety of roles that frequently involve screening for social needs.

### The 10-Item Social Determinants of Health Screening Questionnaire and Referral Procedures

In 2017, a multidisciplinary committee within the health system used the suggested procedures from the Health Leads Social Needs Screening Toolkit [18] to develop a 10-item Social Determinants of Health Screening Questionnaire (Table 1). Clinically validated question items from the toolkit’s screening library were chosen to assess the essential social needs domains (housing instability, utility strain, food insecurity, transportation, financial resources strain, and exposure to violence) and 2 optional social needs domains (childcare and behavioral and mental health). The questionnaire’s reading level was slightly below the sixth-grade reading level (Flesch-Kincaid grade level of 5.9); Microsoft Word Editor was used to calculate readability statistics [19]. To accommodate the language diversity within the health system, the questionnaire was translated into 9 languages.

**Table 1:** Social Determinants of Health Screening Questionnaire.

Screening questions with “yes/no” response options	Social needs domain
Question 1: “Are you worried that in the next two months you might not have a safe place to live? (eviction, kicked out homelessness)”	Housing instability^a^
Question 2: “Are you worried that the place you are living now is making you sick? (has mold, bugs, rodents, water leaks, not enough heat)”	Housing quality^b^
Question 3: “In the last 3 months, has the electric, gas, oil or water company threatened to shut off services to your home?”	Utility strain^a^
Question 4: “In the last 12 months, did you worry that your food could run out before got money to buy more?”	Food insecurity^a^
Question 5: “In the last 3 months, has lack of transportation kept you from medical appointments or getting your medication?”	Transportation^a^
Question 6: “In the last 3 months, did you skip buying medication or going to the doctor’s appointment to save money?”	Financial resources strain^a^
Question 7: “Do you need help getting childcare or care for an elderly or sick adult?”	Childcare^c^
Question 8: “Do you need legal help? (child family services, immigrations, housing, discrimination, domestic issues, etc.)”	Legal^b^
Question 9: “Are you finding it so hard to get along with a partner, spouse, or family member that it is causing you stress?”	Behavioral and mental health^c^
Question 10: “Does anyone in your like hurt you, threaten you, frighten you or make you feel unsafe?”	Exposure to violence^a^

^a^This domain is identified as an essential social needs domain in the Health Leads Toolkit [18].

^b^This domain is identified as an important social needs issue by the multidisciplinary committee of the health system.

^c^This domain is identified as an optional social needs domain in the Health Leads Toolkit [18].

The 10-item Social Determinants of Health Screening Questionnaire was integrated into the EHR across the entire health system in 2018. The health system uses paid, trained, and supervised community members to provide health education and coaching, assistance with clinical services, and community resource connections [20]. However, practice sites within the system determine their own procedures for using the EHR Social Determinants of Health Screening Questionnaire and criteria for community health worker referrals. The practice site that participated in this pilot project had not incorporated a systematic procedure for administering the Social Determinants of Health Screening Questionnaire during clinical procedures by the onset of the COVID-19 pandemic in early 2020.

### Plan-Do-Study-Act Framework

The Plan-Do-Study-Act framework [6] was used to inform how we developed and evaluated the project, which was designed to help the practice site with social needs screening and to provide real-world, service-based learning for preclinical medical student volunteers during the COVID-19 shutdown. Our process steps are outlined below.

#### Plan

Third-year medical students collaborated with the medical director, social worker, and community health worker from the clinical site to develop the project procedures and training program. The planning process addressed procedures for conducting the telephone interviews, entering patient responses into the EHR, and referring patients with social needs to the social worker and community health worker.

#### Do

Preclinical, second-year medical student volunteers were recruited by email. They participated in a 2-hour skills development orientation that taught them how to navigate the Epic platform EHR, screen for social needs, refer patients with social needs, and approach sensitive topics empathetically. The third-year medical students provided ongoing mentoring, peer support, and guidance (ie, they answered questions) for contacting patients via telephone and conducting patient interviews. The preclinical students conducted 47 social needs screening phone interviews and referred 20 patients.

#### Study

We evaluated screening outcomes by using the data extracted from the patients’ EHRs as well as project-specific data. Students’ feedback was obtained to evaluate the pilot project and to elicit recommendations for using service-based learning as a modality for increasing preclinical students’ knowledge and skills related to social determinants of health.

#### Act and Adjust

The lessons learned from this pilot project are being used to inform curriculum planning for preclinical courses that address social determinants of health. The project findings provide insights for future quality improvement initiatives and research that focuses on social needs within the context of health care.

### Social Determinants of Health Screening Telephone Calls

Social determinants of health screening was performed by the preclinical medical students via telephone; the calls were conducted in patients’ preferred language (English or Spanish). Bilingual students called patients who preferred to communicate in Spanish. Our feasibility case study focused on screening calls conducted during May and June 2020. All calls were made by using clinic-approved cellphone apps (eg, Doximity [Doximity Inc]) affiliated with the clinic’s telephone number. Patients responded to the ten social risk questions by answering “yes” or “no” to each of the items in the screener. Patients who answered “yes” to any item were referred to an appropriate care provider. Referrals were made to a trained and supervised community health worker, who was an embedded staff member of the participating practice site. The site’s community health worker linked patients and their households to the appropriate community, state, and federal resources. For patients who preferred to communicate in Spanish, the community health worker, who was not bilingual, used a telephone translation service. Any patient who expressed any safety concerns (eg, domestic violence) or exhibited relationship stress was referred directly to a social worker for assistance and was not included in the analysis.

### Data Extraction and Institutional Review Board Approval

For this project, the social determinants of health screening data were extracted by an analytics team and provided to administrative and medical directors within the ambulatory network for quality improvement investigation–related activities. These data included patients’ responses to the Social Determinants of Health Screening Questionnaire; dates of visit encounters; and patients’ medical record numbers, which facilitated linkages to other databases. This project was reviewed and approved by the Montefiore-Einstein Institutional Review Board (approval number: 2017-8434).

### Primary Outcomes and Covariates

The primary outcomes for this project were the number of screenings completed and referral status (ie, patients’ uptake of social services that were recommended by the community health worker). Completed screenings were defined as screenings in which patients answered all 10 questions. Community health worker referrals were categorized as either successful or unsuccessful. A referral was successful if the community health worker helped a patient access at least 1 resource related to a screened social need. If a patient had self-identified social needs but the community health worker encounter did not facilitate access to relevant resources, the referral was categorized as an unsuccessful referral. Independent covariates were extracted from the REDCap (Research Electronic Data Capture; Vanderbilt University) database [21] based on patients’ self-reports and included sex; age; race and ethnicity; preferred spoken language; and social need categories, including housing needs, benefit needs, youth and family service needs, legal needs, and “other” needs. Comments in REDCap were used to describe needs in the “other” category. These needs were not specific, and these comments included the term *social need* or *community resource*.

## Results

### Questionnaire and Demographic Data

Questionnaire and demographic data were retrieved via Epic. Hispanic and non-Hispanic Black patients constituted the majority of patients (37/45, 82%). Of the 45 patients who had screening data, 20 had at least 1 social need. Almost all of the patients with a social need (19/20, 95%) were referred to the community health worker, who reached most of them (16/19, 84%) to provide a social needs consultation between May and October 2020. Table 2 summarizes the demographic characteristics and outcomes of all patients who completed screening. Patients who were successful in obtaining social needs resources tended to be older than those who were unsuccessful. Spanish-speaking patients constituted 38% (3/8) of the patients who were unsuccessful in obtaining social needs resources.

As shown in Table 3, the majority of patients (13/16, 81%) had 1 social need identified. The most common social need was food insecurity, which was reported by half (8/16, 50%) of the patients who had consultations with the community health worker and was irrespective of whether the referral was successful.

**Table 2:** Social determinants of health screening, community health worker referrals, and success in obtaining needed social service resources.

Patient characteristics		Completed screening (n=45)	No social needs identified (not referred; n=25)^a^	Community health worker referral consultations (n=16)^b,c^	
				Successful in obtaining social service resources (n=8)	Unsuccessful in obtaining social service resources (n=8)
Age (years), mean (SD)		60 (5.7)	60 (5.1)	58 (6.5)	54.5 (6.6)
**Sex, n (%)**					
	Male	19 (42)	13 (52)	2 (25)	1 (13)
	Female	26 (58)	12 (48)	6 (75)	7 (88)
**Race and ethnicity, n (%)**					
	Hispanic	21 (47)	8 (32)	5 (63)	6 (75)
	Non-Hispanic Black	16 (36)	12 (48)	1 (3)	2 (25)
	Non-Hispanic White	2 (4)	1 (4)	1 (3)	0 (0)
	Non-Hispanic	1 (2)	0 (0)	1 (3)	0 (0)
	Asian or Pacific Islander	1 (2)	1 (4)	0 (0)	0 (0)
	Other or unknown	4 (9)	3 (12)	0 (0)	0 (0)
**Preferred spoken language, n (%)**					
	English	34 (69)	21 (84)	4 (50)	5 (62)
	Spanish	14 (31)	4 (16)	4 (50)	3 (38)

^a^Patients who responded to all of the social needs questions were not referred to the community health worker.

^b^A total of 3 patients were lost to follow-up or did not report having social needs during the community health worker assessment; therefore, they were not included in the analysis.

^c^One patient expressed concerns regarding personal safety, was referred to the social worker rather than the community health worker, and was excluded from the analysis.

**Table 3:** Community health worker consultations.

Social needs		Patients who were successful in obtaining social service resources (8/16, 50%), n (%)	Patients who were unsuccessful in obtaining social service resources (8/16, 50%), n (%)
**Number of social needs**			
	1	7 (88)	6 (75)
	2	0 (0)	1 (13)
	3	1 (13)	1 (13)
**Type of social needs**			
	Food insecurity	4 (50)	4 (50)
	Housing^a^	1 (13)	2 (25)
	Other needs	4 (50)	5 (63)

^a^This need was related to applying for a bed in a shelter or for public housing.

### Students’ Feedback

The second-year preclinical student volunteers indicated that their motivation for participating in this project came from the desire to do something concrete and useful during the COVID-19 pandemic and the recognition of their lack of clinical experience and limited capacity to help in clinical settings. Students’ feedback also addressed how the experience helped to expand their clinical skills, as indicated in the following comment:

…there is a piece of your education that has to be about how you talk about social determinants of health…but there is another piece where now that you’ve identified a need, what are you going to do...Those are two different skill sets and I think I got practice with both of those things by doing the calls.

The preclinical student volunteers supported adding more service-based initiatives and more formal social determinants of health training to the curriculum. One comment focused on how aspects of their medical education were both similar to and different from their real-world project experience, as follows:

We do a lot of talking [in medical school] about what are the social determinants of health…but there are gaps in understanding what the particular needs are in a particular community and talking to patients about them, which can be really tough. It is kind of like taking a sexual history. The first time you do it, it feels very awkward and invasive…you really have to do it multiple times to learn how to do it well…often maybe we are doing it [asking social needs questions] for the first time as third years….

## Discussion

### Main Results

The preclinical medical student volunteers indicated that participating in this service-based learning project increased their ability to assess social needs and make social service referrals by using the EHR 10-item Social Determinants of Health Screening Questionnaire. Of the patients with a social need, 80% (16/20) had a consultation with the community health worker, and half of these patients (8/16, 50%) were connected to at least 1 resource to address their social needs. About one-third (14/45, 31%) of the total patients who were screened preferred to speak Spanish, but Spanish-speaking patients constituted 38% (3/8) of the patients who were unsuccessful in obtaining social needs resources after their community health worker consultation. All of the community health worker consultations with patients who preferred to communicate in Spanish were conducted via a translation service because the community health worker was not a Spanish speaker.

### Comparison With Prior Work

Fiori et al [2,3] used a similar social determinants of health survey and reported a successful social service uptake (ie, social services recommended by the community health worker) rate of 43%; the screening surveys were completed in the waiting rooms of a pediatric ambulatory clinic, and the primary care physician reviewed the surveys with the patients. Although addressing the five major social determinants of health (food security, housing access, transportation issues, utility needs, and interpersonal violence) can improve patient outcomes, a 2019 paper by Fraze et al [4] reported that only about 16% of physician practices in the United States screen for all 5 domains. Medical training needs to address how to integrate an empathic discussion of social needs as a standard of care. Taking a sexual history in an inoffensive way is generally taught via practice interviews with standardized patients, and this approach may be applicable to assessing the social determinants of health. Didactics and service activities may facilitate the teaching of needed skills. Screening for determinants of health can be incorporated into community-based service learning programs.

The feedback from the student volunteers suggests that undergoing preparatory training, administering the survey, and making community health worker referrals were valued as service-based learning experiences in health disparity intervention. The evaluation of medical students’ learning experiences can provide insights for skills training in undergraduate medical education. Medical students who learn about the social determinants of health through service and formal curricula have been explored in the literature [22,23]. The Health Scholars Program was an immersive 9-month pilot curriculum on the social determinants of health in which medical students and other types of health professional students in Pennsylvania learned through community service, didactics, and critical reflection [20]. Additionally, medical students at the Morehouse School of Medicine in Atlanta, Georgia, participated in a medical-legal, 4-session curriculum that aimed to teach students how to collaborate in medical-legal partnerships to identify and address the social needs of patients in their communities [21]. In both cases, there was a significant pre-post increase in medical students’ desire to screen for social needs [22,23]. There is growing evidence that providing undergraduate medical students with tools for screening and tracking social determinants of health via the EHR acculturates them to the importance of addressing social determinants of health to reduce health disparities [9]. Individual- and community-level social determinants (ie, those tracked in the EHR) have been proposed as vital signs [24]. The EHR infrastructure and a trained and motivated workforce provide the foundation needed to adequately address social needs and reduce health disparities.

### Limitations

There are several limitations to this case study. First, the Plan-Do-Study-Act cycle focused on immediate needs and did not address how to integrate social determinants of health screening into the clinical routine. Second, the patient sample size was too small for statistical comparisons. Third, success was defined as a patient being connected with at least 1 social needs resource; therefore, if a patient had more than 1 social need and only 1 was addressed, their encounter was deemed successful, even if their needs were only partially addressed. Fourth, medical students entered social needs screening information into the EHR, and referral data were recorded in REDCap. As such, errors and misclassification bias are possible. Further, some patients may have underreported social needs due to perceived stigma, the sensitive nature of certain topics, or disclosure concerns, thus raising the possibility of self-report bias. Fifth, the community health worker and some of the medical students involved in this study were not Spanish speakers and needed to use a phone interpreter to interact with Spanish-speaking patients, which could have affected rapport building and the exchange of information. Sixth, the student volunteers represented a little over 5% (11/180, 6.1%) of the average number of enrolled students, and methods for increasing the proportion of medical students who participate in service-based learning for social needs assessment should be considered. Finally, our qualitative data were limited to informal feedback from the medical students, and we did not systematically ask the students, patients, or the community health worker to reflect on the process or the lessons learned. Despite these limitations, our findings may provide useful information that can inform the planning of medical education, health system research, and quality improvement initiatives.

### Conclusions

The students developed proficiencies in assessing social needs and documenting their assessments and related referrals in the EHR. In this successful service-based learning experience, preclinical medical students learned how to use community health worker referrals to address social needs. The participating students also gained experience in broaching potentially uncomfortable topics, identifying related needs, and performing the appropriate next steps to address these needs. Since the participating second-year medical students were in preclinical training, this volunteer experience may have been their first exposure to an ambulatory setting and social needs assessment. Opportunities for asking patients about their struggles with social needs can provide a meaningful and memorable experience to medical student trainees. Early practical exposure to social needs assessment has the potential to help medical students develop the ability to address social concerns prior to entering clinical clerkships in the third year of medical school. Finally, medical students gained familiarity with the EHR prior to entering third-year clerkships. Integrating social determinants of health into undergraduate medical curricula could increase the awareness of social needs in the physician workforce, and the integration of the 10-item Social Determinants of Health Screening Questionnaire has resulted in a tool for integrating social needs assessment into clinical practice. Physicians, who are aware of social needs and have the electronic medical record tools and staff resources needed to act, can create workflows to make social needs assessment and services integral components of health care. Larger-scale studies need to assess the effect of integrating screening for social needs and connecting patients to the appropriate social services into routine primary care procedures.

## Appendix

Multimedia Appendix 1 Social determinants of health screening project: preclinical student volunteer training (PowerPoint slides).

## Abbreviations

AAMC: Association of American Medical Colleges
EHR: electronic health record
REDCap: Research Electronic Data Capture
